# Latent class models for *Echinococcus multilocularis* diagnosis in foxes in Switzerland in the absence of a gold standard

**DOI:** 10.1186/s13071-017-2562-1

**Published:** 2017-12-19

**Authors:** Belen Otero-Abad, Maria Teresa Armua-Fernandez, Peter Deplazes, Paul R. Torgerson, Sonja Hartnack

**Affiliations:** 1 Section of Veterinary Epidemiology, Vetsuisse Faculty, University of Zurich, Zurich, Switzerland; 2 Institute of Parasitology, Vetsuisse Faculty, University of Zurich, Zurich, Switzerland; 3 Laboratorio de Vectores y Enfermedades transmitidas, Facultad de Veterinaria, CENUR Litoral Norte - Salto- Universidad de la República, Rivera 1350, 50000 Salto, Uruguay

**Keywords:** *Echinococcus multilocularis*, Foxes, Diagnostic test, Diagnostic sensitivity, Diagnostic specificity

## Abstract

**Background:**

In Europe the principal definitive host for *Echinococcus multilocularis*, causing alveolar echinococcosis in humans, is the red fox (*Vulpes vulpes*). Obtaining reliable estimates of the prevalence of *E. multilocularis* and relevant risk factors for infection in foxes can be difficult if diagnostic tests with unknown test accuracies are used. Latent-class analysis can be used to obtain estimates of diagnostic test sensitivities and specificities in the absence of a perfect gold standard. Samples from 300 foxes in Switzerland were assessed by four different diagnostic tests including necropsy followed by sedimentation and counting technique (SCT), an egg-PCR, a monoclonal and a polyclonal copro-antigen ELISA. Information on sex, age and presence of other cestode species was assessed as potential covariates in the Bayesian latent class models. Different Bayesian latent-class models were run, considering dichotomized test results and, additionally, continuous readings resulting in empirical ROC curves.

**Results:**

The model without covariates estimated a true parasite prevalence of 59.5% (95% CI: 43.1–66.4%). SCT, assuming a specificity of 100%, performed best among the four tests with a sensitivity of 88.5% (95% CI: 82.7–93.4%). The egg-PCR showed a specificity of 93.4% (95% CI: 87.3–99.1%), although its sensitivity of 54.8% was found moderately low (95% CI: 48.5–61.0%). Relatively higher sensitivity (63.2%, 95% CI: 55.3–70.8%) and specificity (70.0%, 95% CI: 60.1–79.4%) were estimated for the monoclonal ELISA compared to the polyclonal ELISA with a sensitivity and specificity of 56.0% (95% CI: 48.0–63.9%) and 65.9% (95% CI: 55.8–75.6%), respectively. In the Bayesian models, adult foxes were found to be less likely infected than juveniles. Foxes with a concomitant cestode infection had double the odds of an *E. multilocularis* infection. ROC curves following a Bayesian approach enabled the empirical determination of the best cut-off point. While varying the cut-offs of both ELISAs, sensitivity and specificity of the egg-PCR and SCT remained constant in the Bayesian latent class models.

**Conclusions:**

Adoption of a Bayesian latent class approach helps to overcome the absence of a perfectly accurate diagnostic test and gives a more reliable indication of the test performance and the impact of covariates on the prevalence adjusted for diagnostic uncertainty.

**Electronic supplementary material:**

The online version of this article (10.1186/s13071-017-2562-1) contains supplementary material, which is available to authorized users.

## Background

*Echinococcus multilocularis* is a zoonotic tapeworm found in the northern hemisphere and mainly transmitted between foxes and small mammals [1]. Humans are accidental hosts that can become infected through the oral intake of parasite eggs. In the absence of treatment, potentially fatal alveolar echinococcosis (AE) develops [2]. There is evidence of a geographical expansion of the known *E. multilocularis* endemic area in Central Europe towards the north, west and east of the continent [1]. Expert consensus foresees a delayed increase in the occurrence of AE cases in Europe within the next decades due to its long incubation period [3]. As a consequence, information on the parasite distribution in the red fox (*Vulpes vulpes*), the principal definitive host in Europe of *E. multilocularis*, is paramount to estimate the potential risk of human infection and assist in prevention efforts [4, 5]. Three of the diagnostic techniques frequently used for *E. multilocularis* detection in the definitive host include the visual identification of adult worms in the small intestine at necropsy through the sedimentation and counting technique (SCT), the parasite coproantigen detection and the amplification of DNA from parasitic eggs present in the fox faeces [6]. The performance of these tests, for a given population, are commonly measured based on their diagnostic sensitivity and specificity. The necropsy followed by SCT is considered the reference test with a very high specificity (around 99%), as the morphological features of *E. multilocularis* allow an unequivocal diagnosis in most cases [7]. However, some limitations concerning SCT’s sensitivity must be taken into consideration [8, 9], as high worm burdens are required. Despite some available modifications in its performance [10, 11], this technique remains laboratory intensive, time-consuming and expensive, and entails the implementation of strict safety precautions to minimize the risk of infection of the personnel involved. Also, this procedure requires the collection of dead red foxes limiting its practicality for population studies. The detection of parasite antigens in the fox faeces through the binding of antigen-antibody in an enzyme-linked immunosorbent assay (ELISA) remains an alternative method for the diagnosis of parasite infection in foxes. The coproantigen test has the advantage of detecting also pre-patent infections [12–14]. Polyclonal- and monoclonal-antibody-based ELISAs have been developed for the detection of E. multilocularis [12, 13, 15, 16]. High sensitivities (80–95%) and specificities (≈ 0–99%) have been originally reported for the coproantigen test [12, 13] although sensitivities are strongly dependent on fox worm burdens [13, 17–19]. Being a relatively safe, rapid and inexpensive test, it qualifies as a potential technique for mass screening in the fox population from endemic areas where false positives are acceptable. The parasite distribution is known to be skewed with a small number of foxes harbouring a high number of worms [20]. It is believed that foxes with moderate to high worm burdens might contribute to most of the environmental contamination and hence, to human exposure [21]. Thus, it is paramount that the diagnostic test could adequately identify them. Consequently, the present study included a scenario where foxes were harbouring worm loads of 100 or more parasites to evaluate the potential performance of one of the coproantigen test for population studies. A third diagnosis option is the detection of *E. multilocularis* genetic material excreted with the faeces of the definitive host through the amplification by the polymerase chain reaction (PCR). Since the first publication of this technique for *E. multilocularis* diagnosis [22] different approaches have been developed to improve its performance on faeces [23–31]. This method is highly specific, but low worm burdens and the presence of inhibitory components may compromise its sensitivity [29, 32]. However, these limitations might be overcome by the development of newly magnetic capture-PCR and the implementation of real-time PCR procedures assigning this diagnostic procedure with a sensitivity comparable to SCT’s [9]. Nevertheless, it remains a labour intensive and expensive technique, so its application in population studies is commonly restricted as a confirmatory test for coproantigen positive samples [13, 14, 26]. Despite several available *E. multilocularis* diagnosis options in foxes none of them can be regarded as a perfect gold standard test, with 100% specificity and 100% sensitivity. Therefore, prevalence studies in foxes rely on imperfect diagnostic methods and these limitations in tests’ accuracies should be taken into account when reporting and interpreting their results [6].

A widely used approach to overcome the lack of a perfect gold standard test is through the application of latent class models, using frequentist or Bayesian methods. Hui & Walter [33] originally described the latent class models using a frequentist approach by first considering the case where two tests were applied to two populations with different prevalences, under the assumption of sensitivities and specificities being constant across populations and conditional independence between the two tests. Hui & Walter also showed that given the model assumptions are met if the condition of S ≥ R / (2 R-1 – 1) is satisfied, where S represents the number of populations and R the number of tests applied, there will be enough degrees of freedom to estimate the parameters of interest. Since then, derivations of the Hui & Walter model have been developed to estimate the unknown parameters that are latent in the data when a standard gold test is not available [34]. When Bayesian approaches are implemented prior information can be incorporated and potentially conditional dependencies assessed. The evaluation of the accuracy of the diagnostic methods for *E. multilocularis* detection by latent class analysis has become increasingly common [9, 31, 35].

Here, we applied Bayesian latent class models using the results of four diagnostic tests for *E. multilocularis* in foxes, the necropsy and SCT, the monoclonal ELISA, the polyclonal ELISA and the egg-PCR, to a single reference fox population in Switzerland aiming to address the following research questions: (i) what is the true parasite prevalence?; (ii) what are the performance characteristics of the diagnostic tests?; (iii) have any of the three covariates assessed (fox age, sex and presence of co-infection with other cestodes) an effect on the true infection status?; (iv) do any differences exist between the selection of the cut-off point for the ELISA by adopting Bayesian latent class models compared with the employment of the classic method of considering the necropsy and SCT as the gold standard test?; (v) has the selection of the ELISA cut-off point any effect on the estimation of performance of the other tests?; and (vi) what is the impact on the performance of the monoclonal-ELISA if we change the threshold for the necropsy and SCT results to be considered a sample positive only with 100 or more *E. multilocularis*?

## Methods

### Fox samples

A total of 300 red foxes (*Vulpes vulpes*) were examined at the Parasitology Institute, University of Zurich, for *E. multilocularis* as part of the European Research Programme on Emerging and Major Infectious Diseases of Livestock (EU-Project EMIDA). The animals were shot and collected by hunters at different locations in the midlands of Switzerland during the official hunting seasons between 2012 and 2014. Thus, it is representative of this area and not of the alpine regions, which tend to have a lower prevalence of infection. According to the Swiss Animal Welfare act, article 3, this research project is not considered as an animal experiment. Due to the risk associated with the handling of infectious materials, a fraction of the small intestines retrieved from the fox carcasses was frozen at -80 °C for 5 days before proceeding with their parasitological examination [36]. However, 163 of them were only kept at 4 °C as there was a need to collect viable *E. multilocularis* eggs for experimental infection of rodents in the context of the EMIRO project, a research project in the framework of the EMIDA ERA-NET [37].

### Diagnostic tests

Four diagnostic procedures were performed for each fox. The original data file with the diagnostic test results and information of covariates can be found in the Additional file 1: Table S1.

### Necropsy and sedimentation counting technique (SCT)

The small intestines were removed during the necropsy of the fox carcasses to be later used for the identification of adult stages of *E. multilocularis* by SCT. This procedure was carried out as previously described in [20]. The suggested sensitivity of this procedure is 98% [38]. Results were recorded for fox classification as positive (1) or negative (0) for *E. multilocularis* presence. During necropsy, information related to the sex of the fox, presence of other cestode species and fox age was recorded for each animal. This information was registered by assigning numerical values of 0 and 1 as follows: female = 0 and male = 1; young = 0 and adult = 1; and absence of cestodes = 0 and presence of cestodes = 1. The proportion of foxes by age, sex and presence of cestodes coinfection are displayed in Table 1. The age determination of the fox was roughly estimated based on the displaying level of tooth wear [39]. Animals with front upper incisors showing a sharp and visible fleur-de-lys pattern were regarded as young foxes (< 1 year-old) while animals displaying a high degree of attrition were classified as adults (> 1 year old). Also, fresh faecal samples were collected from the rectum of each fox and kept at -80 °C for at least 1 week before being processed.

**Table 1:** Observed proportions of collected foxes by age, sex and presence of cestodes co-infection

		Sex		Total
		Female	Male	
Age	Young	0.21	0.19	0.40
	Adult	0.25	0.35	0.60
	Total	0.46	0.54	1^a^
Cestodes	Yes	0.25	0.36	0.61
	No	0.21	0.18	0.39
	Total	0.46	0.54	1^a^

^a^Total number of foxes = 300

### Coproantigen enzyme-linked immunosorbent assay (ELISA)

Part of the faecal samples was analysed using two coproantigen tests, specific for *E. multilocularis* diagnosis. Both ELISAs have been produced by the Institute of Parasitology of Zurich: the polyclonal antibodies based ELISA (pAb- ELISA) using rabbit and chicken egg antibodies was performed as described [13] and the recently modified monoclonal antibody-based ELISA (mAb-ELISA) using a rat monoclonal antibody directed against *E. multilocularis* integument antigen and rabbit antibodies as described [40]. The ELISA results were expressed in corrected A405nm reading values obtained from the subtraction of the specific reaction minus the unspecific reaction [40]. The original overall reported sensitivity of the pAb-ELISA, calculated as the mean A405nm reading value plus three times the standard deviation of faecal samples or intestinal contents of *Echinococcus*-free dogs and foxes, was 84%, strongly dependent on worm burdens [13]. The ELISA results were classified as positive (1) or negative (0) considering the necropsy and subsequent SCT as the perfect gold standard test. The receiver operating characteristic (ROC) curve was built by comparing the ELISA’s numerical continuous reading values to the dichotomous necropsy and SCT results by using the *pROC* R package [41].

### Copro-DNA detection by multiplex polymerase chain reaction (egg-PCR)

The remainder of the faecal material was used for the isolation and microscopy identification of taeniid eggs as described in [24], followed by egg-DNA extraction and egg-DNA detection by a multiplex PCR following indications of [27].

The originally proposed sensitivity for this procedure, estimated by comparison with the results derived from the microscopic examination of the deep intestinal mucosal scrapings after necropsy, was 89% dependent on worm burdens and the maturity of the worms [25]. The combination of egg isolation and egg-DNA detection by PCR gave the information to classify the samples as positive (1) or negative (0) for *E. multilocularis* infection.

### Bayesian latent class models

The test results on *E. multilocularis* infection in foxes were analysed using latent class models within the Bayesian framework described in detail in [42]. This approach aims to identify appropriate models, which jointly estimate the diagnostic test accuracies, conditional dependencies and disease prevalence and simultaneously to identify those covariates which are related to the true prevalence (and not solely to the apparent prevalence) in the absence of a true gold standard. The probability model used is the binomial distribution to model prevalence. The description of the Bayesian latent-class model code used for the analysis of three and four diagnostic tests is available in the Additional files 2 and 3.

### Latent class analysis of three tests

The first part of the latent class analysis included the results of three of the diagnostic tests including necropsy and SCT, pAb-ELISA and egg-PCR. The model parameters encompassed the true parasite prevalence, the sensitivities and specificities of the three diagnostic tests (Se1, Se2, Se3, Sp1, Sp2 and Sp3) and their corresponding two-way covariance terms. With the aim to adjust for conditional dependencies, first, all potential covariances (σSe12, σSe23, σSe13 and σSp23) were included simultaneously. Subsequently, in the absence of evident covariances (i.e. the posterior mean was equal to zero), they were set to 0. Since the specificity of the necropsy and SCT has been reported to be close to 99% [36] this parameter (Sp1) was fixed to 1.

### Latent class analysis of four tests

The second part of the latent class analysis included the results of the four diagnostic tests, which included necropsy and SCT, pAb-ELISA, mAb-ELISA and egg-PCR. The model parameters encompassed the true parasite prevalence, the sensitivities and specificities of the four diagnostic tests (Se1, Se2, Se3, Se4, Sp1, Sp2, Sp3 and Sp4) and their covariance terms. Once more the specificity of the necropsy and SCT was fixed to 1. Similarly, first, all potential nine covariance terms (σSe12, σSe23, σSe34, σSe13, σSe14, σSe24, σSp23, σSp24 and σSp34) were included simultaneously, and set to 0 subsequently, when the posterior means were equal to zero.

### Model priors

Non-informative beta priors (1,1), as well as informative beta priors, were selected for the latent prevalence and the test sensitivities and specificities, as beta distributions are well suited to describe the uncertainty associated with a binomial probability. The software Betabuster was used to obtain the values for the informative priors based on literature. The informative priors are presented in the Additional file 4: Table S2 and Additional file 5: Table S3. A sensitivity analysis was performed to assess the potential influence of the priors on the posteriors and assess the robustness of the results. The sensitivity analysis consisted of varying the informative priors for each of the parameters of interest, one at a time, while keeping the other priors fixed for both the three- and the four-test models. We varied the informative prior of the parameter of interest systematically from assuming that the parameter is larger than 0.9, 0.8 and so on until 0.1, with a respective mode of 0.95, 0.85 and so on until 0.25. With this approach we obtained a number of informative priors, ranging from strong priors with a small variance (steep curve) or high precision, e.g. “greater than 60 % with a mode at 65%” to rather uninformative priors e.g. “greater than 10% and a mode at 95%” (flat curve). The latter one is close to the uninformative priors dbeta (1,1). Furthermore, with this approach we also obtained some priors which are, potentially, in conflict with our data, e.g. we assume that the sensitivity is not close to 95% or 25%. Results of the sensitivity analysis for the sensitivity of PCR in the three-test model are shown in the Additional file 6. The covariance terms were assumed to be uniformly distributed ranging from -1 to 1.

### Model fitting and comparison

Latent class models were fitted using Markov chain Monte Carlo (MCMC) simulation by employing the free statistical software JAGS version 3.1.0 [43]. For each model, three chains of the Gibbs sampler were run independently for 200,000 iterations after an initial burn-in of 50,000 iterations. The behaviour of the MCMC chains was monitored through the plotting of the posterior values to identify potential converging problems. The output files from the Gibbs sampler were analyzed through the package coda [44] calculating the multivariate potential scale factor within the open source software R [45]. The model comparison of goodness-of-fit to the data was based on three criteria. The first criterion included the histograms resulting from the marginal posterior distribution for each covariance term. If the histograms showed the higher frequencies around 0, and the posterior mean was zero, then it was assumed that this term was negligible and thus, its addition did not improve the model. The second criterion was based on the impact experimented by the parameters estimates and their credibility intervals following the addition of a covariance term. The parameter point estimates were reported as the mean of their marginal posterior distributions. If the parameter estimates did not vary greatly, it indicated the redundancy of adding the extra term to the model. The third criterion was based on the deviance information criterion (DIC), which takes into account the deviance of the posterior mean of the parameters and the effective number of parameters used in the model. The smaller the value of the DIC, the better the model fits the data without overfitting.

### Model with covariate pattern

The three covariates, “sex”, “age” and “presence of other cestodes”, were included in the best model one at a time to explore their potential association with the fox infection status. We used a binomial regression model with a logit link function between the true unknown prevalence and the covariate term including an intercept and a slope. The improvement of the model after adding each covariate was established if there was a significant reduction in the DIC (by at least two units) and depending on the impact on the parameter estimates and accuracies. The covariates were regarded as statistically significant associated with *E. multilocularis* infection when the credibility intervals of the slope (expressed in odds ratio) did not include 1. The three MCMC chains ran independently for 200,000 iterations after a burn-in of 50,000 iterations and the plots of the posterior values for each chain were visually checked to identify potential converging problems and multivariate potential scale factors were obtained.

### The receiver operating characteristic (ROC) curve

The ROC curve describes graphically the ELISA performance by plotting the sensitivity on the y-axis against 1-specificity on the x-axis for many different cut-off points. The area under the ROC curve (AUC) provides an overall measure of the accuracy of the ELISA. We produced first two ROC curves, one for the pAb-ELISA and one for the mAb-ELISA with the model for three tests. Subsequently, two ROC curves for both ELISAs with the four-test model, including the cut-offs estimated from the previous analyses, were generated.

### Bayesian empirical pAb- and mAb-ELISA ROC curves

The ROC curves for the ELISA tests were produced by initially considering the results of three tests, then considering the results of all four tests together. For the analyses including three of the tests, two ROC curves were produced, one curve based on the results of the necropsy and SCT, pAb-ELISA and egg-PCR and the other curve based on the results of the necropsy and SCT, mAb-ELISA and egg-PCR. To that end, a hundred potential cut-off values were obtained from the percentile values of the ELISAs’ optical readings (Specific minus Unspecific), ranging from the 1st to the 100th. For each of these 100 cut-off points, the results of the pAb- and mAb-ELISA were classified as positive or negative. Therefore, a hundred different classifications were obtained for the results of both ELISAs. Next, the best-fitting model (without covariates) was run 100 times using each of these hundred classifications obtained for the results of the ELISA. Afterwards, the estimated values of the sensitivities and specificities for both ELISAs obtained from the model were used to produce the two ROC curves for 100 possible cut-off points. Next, the same procedure was carried out to produce the ROC curves for the ELISAs, but now the results of all four tests were included in the analysis. Also, this time the value used to classify the results of the ELISA were the best cut-off determined in the previous three-test models.

### Bayesian empirical mAb-ELISA ROC curve after changing the threshold for the necropsy and SCT

Finally, we changed the threshold criteria for the necropsy and SCT results by assigning a positive value only to the fox samples where 100 or more parasites were counted. The best-fitting model (without covariates) to the results of the four diagnostic tests was run a hundred times, following the same procedure as above, to produce a new mAb-ELISA ROC curve.

## Results

### Bayesian latent class models for three diagnostic tests

Since the posterior means of the three sensitivity covariance terms were distinct from zero, they were included in the final model and are presented in the Additional file 7: Table S4. In contrast, due to the absence of evident covariance (posterior mean equal to zero), the specificity covariance between PCR and pAb-ELISA was set to 0. The addition of sensitivity covariance terms compared to the independence model, without any covariances included led to a decrease of approximately 2% points in the posterior means.

**Fig. 1:**
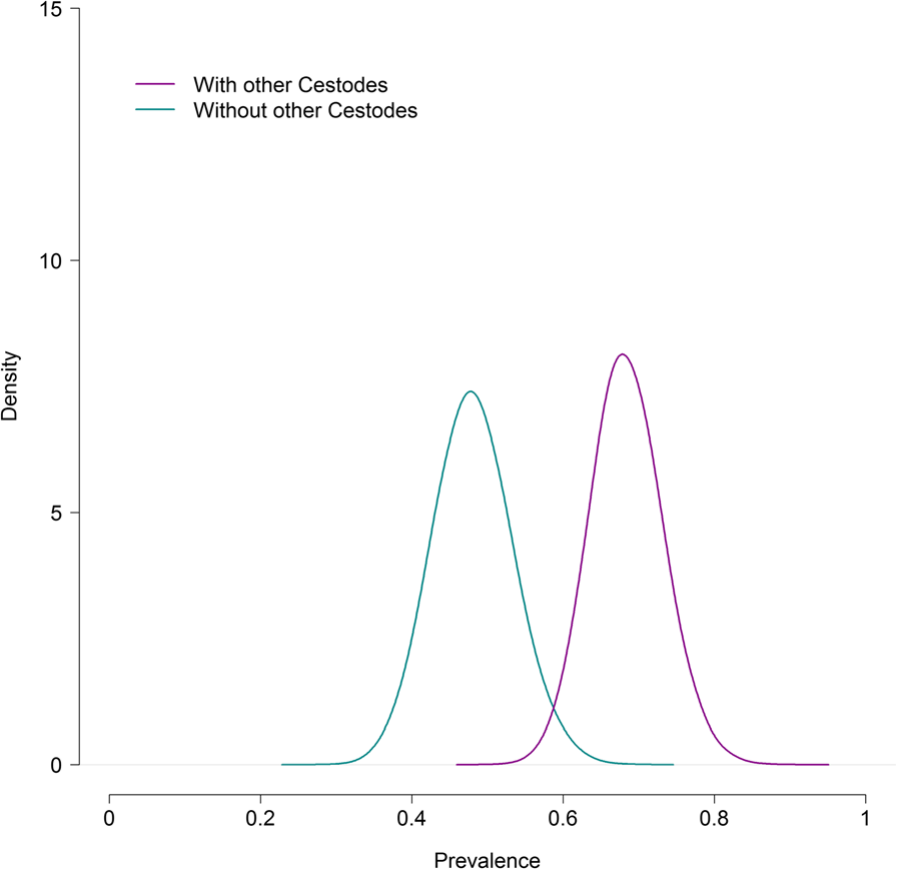
Posterior distribution of *E. multilocularis* prevalence in foxes with and without the significant covariate, “cestodes” for the best-fitting model to the results of three diagnostic tests

**Fig. 2:**
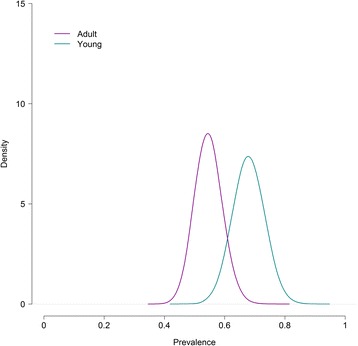
Posterior distribution of *E. multilocularis* prevalence in foxes with and without the significant covariate “age” for the best-fitting model to the results of three diagnostic tests

The estimated parameter values with their 95% credibility intervals and DIC for the best-fitting model with and without covariates are presented in Table 2. Figures 1 and 2 show estimated true *E. multilocularis* prevalence in foxes with and without the significant covariates, “cestodes” and “age”.

**Table 2:** Parameters estimates (posterior means) with their corresponding 95% credibility intervals and the model goodness-of-fit to the data of the best model for three tests with and without covariates

	Model	Model with “age”	Model with “cestodes”	Model with “sex”
SCT				
Se1	0.919 (0.857 to 0.961)	0.91 (0.843 to 0.950)	0.905 (0.834 to 0.958)	0.909 (0.840 to 0.958)
Sp1	1^a^	1^a^	1^a^	1^a^
Egg-PCR				
Se2	0.543 (0.474 to 0.610)	0.539 (0.470 to 0.608)	0.533 (0.465 to 0.602)	0.539 (0.470 to 0.608)
Sp2	0.919 (0.850 to 0.982)	0.917 (0.848 to 0.980)	0.914 (0.843 to 0.977)	0.920 (0.848 to 0.984)
pAb-ELISA				
Se3	0.556 (0.475 to 0.637)	0.553 (0.472 to 0.634)	0.543 (0.461 to 0.626)	0.552 (0.470 to 0.633)
Sp3	0.641 (0.540 to 0.732)	0.638 (0.533 to 0.735)	0.624 (0.512 to 0.723)	0.636 (0.528 to 0.734)
Prevalence	0.584 (0.526 to 0.645)	na	na	na
Cov = 1^b^	na	0.682 (0.577 to 0.783)	0.480 (0.379 to 0.589)	0.587 (0.487 to 0.691)
Cov = 0^b^	na	0.546 (0.305 to 0.775)	0.686 (0.456 to 0.856)	0.613 (0.387 to 0.807)
Intercept	na	0.76 (0.31 to 1.28)	−0.08 (−0.49 to 0.36)	0.35 (−0.05 to 0.80)
Slope (OR)^c^	na	0.56 (0.32 to 0.96)	2.36 (1.37 to 4.16)	1.12 (0.67 to 1.87)
DIC	1129.2	1126.7	1120.4	1130.9

^a^Specificity of necropsy fixed to 1

^b^Prevalence for respective covariate = 1 (adult, with other cestodes and male) and covariate = 0 (young, without other cestodes and female)

^c^Odds ratio

*Abbreviations: Se* sensitivity, *Sp* specificity, *Egg-PCR* polymerase chain reaction, *pAb-ELISA* polyclonal enzyme-linked immunosorbent assay (cut-off determined by considering necropsy and SCT as the gold-standard test), *DIC* deviance information criterion, *na* not applicable

Two covariates, “cestodes” and “age”, were found significantly associated with *E. multilocularis* occurrence in foxes. The addition of the covariate “cestodes” brought the largest improvement in DIC and suggested that foxes with a concomitant cestode infection had double the odds of presenting *E. multilocularis* compared to foxes without it. The model including the covariate “age” experienced a less remarkable improvement in DIC and implied that adult foxes were less likely to be infected with *E. multilocularis* compared to younger animals. The covariate “sex” was found not significant, with no differences in *E. multilocularis* infection between males and females. The addition of covariates to the model had a negligible influence on the parameter estimates.

### Bayesian latent class models for four diagnostic tests

Similarly to the three-test models, all six sensitivity covariances had posterior means unequal to zero and were therefore included in the final model (Additional file 7: Table S4). In contrast, there was no evidence for covariances between specificities (i.e. posterior mean equal to zero), and all three potential specificity covariances were set equal to 0.

**Fig. 3:**
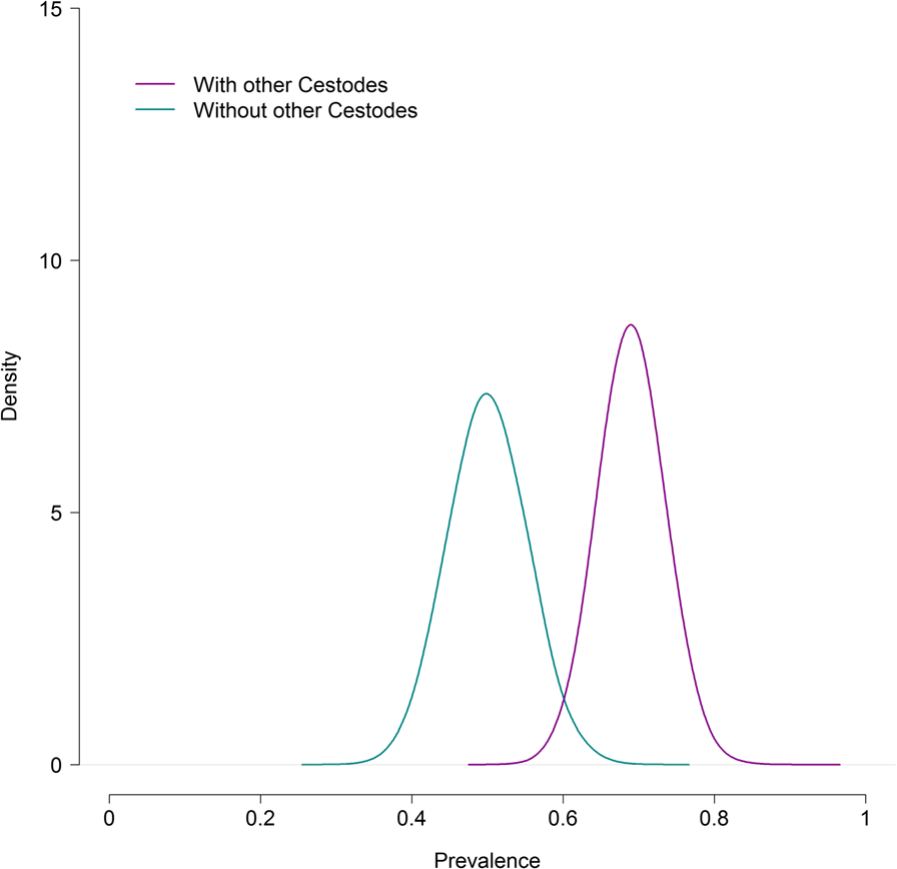
Posterior distribution of *E. multilocularis* prevalence in foxes with and without the significant covariate, “cestodes” for the best-fitting model to the results of four diagnostic tests

**Fig. 4:**
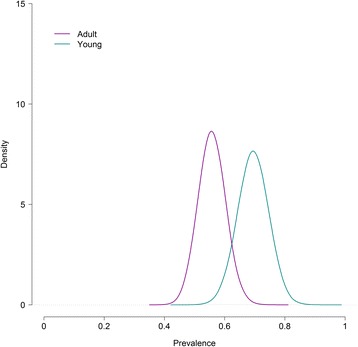
Posterior distribution of *E. multilocularis* prevalence in foxes with and without the significant covariate “age” for the best-fitting model to the results of four diagnostic tests

The parameters estimates with their related 95% credibility intervals and DIC for the best-fitting model with and without covariates are presented in Table 3. Figures 3 and 4 show the *E. multilocularis* prevalence in foxes with and without the significant covariates as well as “cestodes” and “age”.

**Table 3:** Parameters estimates (posterior means) with their corresponding 95% credibility intervals and the model goodness-of-fit to the data of the best model for four tests with and without covariates

	Model	Model with “age”	Model with “cestodes”	Model with “sex”
SCT				
Se1	0.885 (0.827–0.934)	0.879 (0.816 to 0.931)	0.876 (0.811 to 0.930)	0.878 (0.814 to 0.930)
Sp1	1^a^	1^a^	1^a^	1^a^
Egg-PCR				
Se2	0.548 (0.485 to 0.610)	0.544 (0.482 to 0.608)	0.544 (0.482 to 0.606)	0.546 (0.483 to 60.8)
Sp2	0.934 (0.873 to 0.991)	0.936 (0.872 to 0.990)	0.940 (0.874 to 0.992)	0.940 (0.874 to 0.993)
pAb-ELISA				
Se3	0.560 (0.480 to 0.639)	0.558 (0.477 to 0.637)	0.551 (0.471 to 0.631)	0.557 (0.476 to 0.638)
Sp3	0.659 (0.558 to 0.756)	0.659 (0.555 to 0.758)	0.648 (0.540 to 0.749)	0.659 (0.552 to 0.759)
mAb-ELISA				
Se4	0.632 (0.553 to 0.708)	0.629 (0.550 to 0.706)	0.623 (0.544 to 0.701)	0.629 (0.549 to 0.707)
Sp4	0.700 (0.601 to 0.794)	0.701 (0.600 to 0.797)	0.693 (0.590 to 0.791)	0.701 (0.598 to 0.799)
Prevalence	0.595 (0.431 to 0.664)	na	na	na
Cov = 1^b^	na	0.697 (0.594 to 0.794)	0.500 (0.398 to 0.606)	0.596 (0.594 to 0.794)
Cov = 0^b^	na	0.558 (0.312 to 0.784)	0.692 (0.464 to 0.857)	0.631 (0.312 to 0.784)
Intercept	na	0.83 (0.38 to 1.34)	0.00 (−0.04 to 0.43)	0.39 (−0.01 to 0.83)
Slope (OR)^c^	na	0.55 (0.31 to 0.94)	2.24 (1.31 to 3.90)	1.16 (0.96 to 1.96)
DIC	1507.0	1501.9	1497.2	1506.2

^a^Specificity of necropsy fixed to 1

^b^Prevalence for respective covariate = 1 (adult, with other cestodes and male) and covariate = 0 (young, without other cestodes and female)

^c^Odds ratio

*Abbreviations: Se* sensitivity, *Sp* specificity, *Egg-PCR* polymerase chain reaction, *pAb-ELISA* polyclonal enzyme-linked immunosorbent assay, *mAb-ELISA* monoclonal enzyme-linked immunosorbent assay (cut-off for both ELISAs determined by considering necropsy and SCT as the gold-standard test), *DIC* deviance information criterion, *na* not applicable

Once more, the covariates “cestodes” and “age” were found significantly associated with *E. multilocularis* presence in the fox. Again, the model including the covariate “cestodes” displayed the lowest DIC indicating that the odds of *E. multilocularis* infection doubled in foxes with concurrent cestode infection in comparison to foxes without it. The covariate “age” was also found significant although its addition to the model did not cause a remarkable reduction in the DIC. The model suggested lower odds of *E. multilocularis* infection in adults than younger foxes. The covariate “sex” was found not significant, with no differences in *E. multilocularis* infection between male and female foxes. The addition of covariates to the model did not change the parameter estimates.

### The receiver operating characteristic (ROC) curve results

#### Bayesian empirical pAb-ELISA ROC curve from the three-test model

**Fig. 5:**
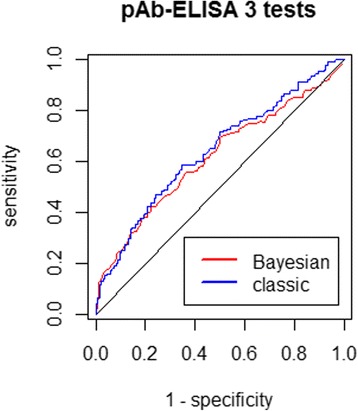
Polyclonal ELISA (pAb-ELISA) ROC curves produced using the classical and the Bayesian approach

The best cut-off point obtained from the pAb-ELISA ROC curve using the classical method of considering necropsy and SCT as a gold standard test was 0.21, assigning the coproantigen test with 58.5% sensitivity, 65.4% specificity and an overall accuracy of 63.8% (95% CI: 57.6–70.1%) given by the AUC. The optimal cut-off value from the Bayesian pAb-ELISA ROC curve using the three-test model was 0.29, assigning the coproantigen test with 42.2% sensitivity, 77.8%, specificity and an overall accuracy of 60.7% given by the AUC. Figure 5 shows both pAb-ELISA ROC curves derived using the classical and the Bayesian approach.

#### Bayesian empirical mAb-ELISA ROC curve from the three-test model

**Fig. 6:**
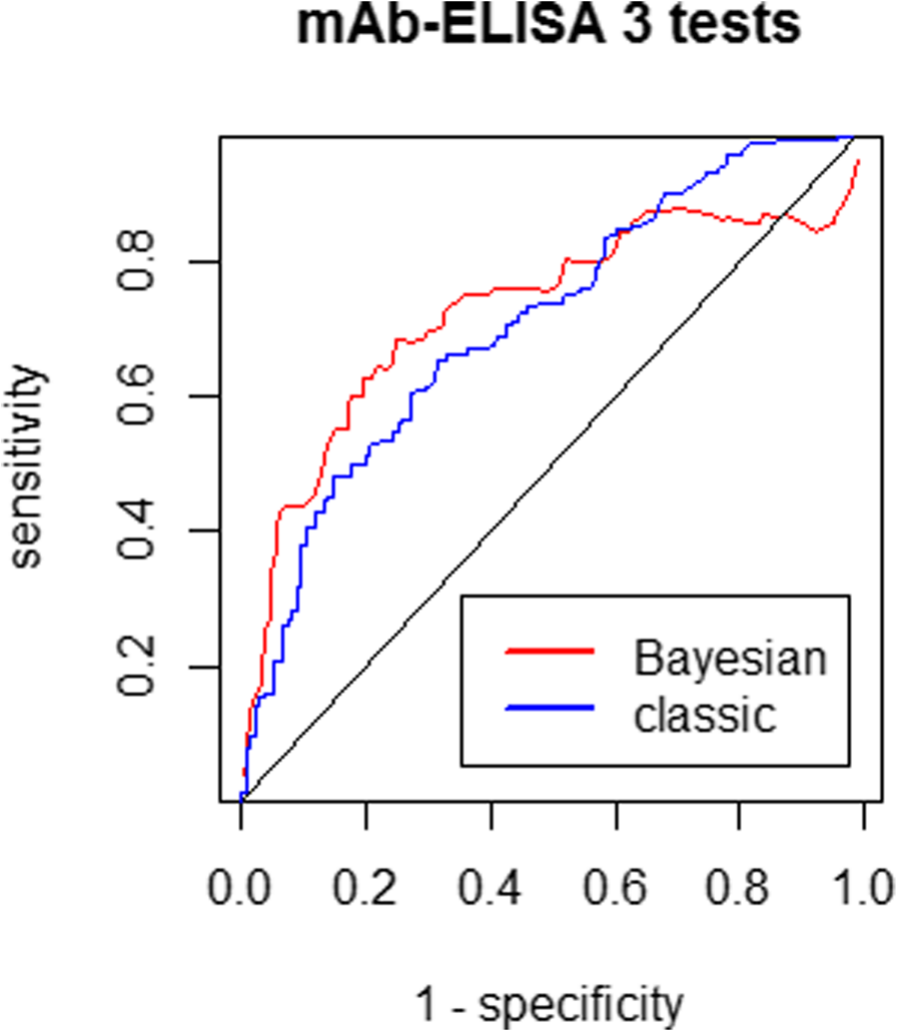
Monoclonal ELISA (mAb-ELISA) ROC curves derived using the classical and the Bayesian approach

The best cut-off point obtained from the mAb-ELISA ROC curve using the classical method was 0.10, assigning the coproantigen test with 65.2% sensitivity, 68.4% specificity and an overall accuracy of 71.2% (95% CI: 65.4–77.0%) given by the AUC. The optimal cut-off value from the Bayesian mAb-ELISA ROC curve using the three-test model was 0.16, assigning the coproantigen test with 68.3% sensitivity, 75.3% specificity and an overall accuracy of 71.7% given by the AUC. Figure 6 shows both mAb-ELISA ROC curves derived using the classical and the Bayesian approach.

#### Bayesian empirical pAb- and mAb-ELISA ROC curves from the four-test model

When including the mAb-ELISA cut-off based on a Bayesian approach in the four-test model, AUC for the pAb-ELISA ROC curve was similar to the three-test model, e.g. 60.7%. The highest sum of the sensitivity plus specificity was 1.20 with an associated sensitivity and specificity of 69.9 and 50.6%, respectively. The corresponding cut-off was 0.17. The second highest sum of sensitivity and specificity was 1.198 with the same cut-off as in the three-test model of 0.29. For this cut-off, the sensitivity and specificity were 41.6 and 78.3%, respectively.

When including the pAb-ELISA cut-off based on a Bayesian approach in the four-test model, the AUC for the mAb-ELISA ROC curve was 76.2% for the same cut-off 0.16 with associated sensitivity and specificity of 70.5% and 80.0%. In Additional file 8: Figure S1 and Additional file 9: Figure S2, ROC curves for both ELISAs with the classical and the Bayesian approach are shown.

**Fig. 7:**
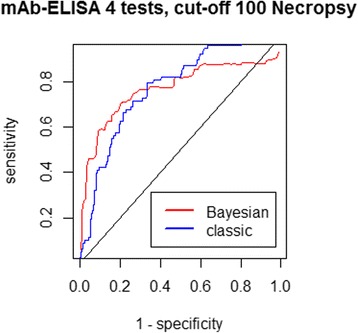
Bayesian monoclonal ELISA ROC when the criteria to be positive by necropsy and SCT is to present 100 or more *E. multilocularis*

The variation of the cut-off points for the classification of both ELISA tests, pAb- and mAb-ELISAs, had virtually no impact on the estimations of the other parameters of interest. The analysis was performed once more using the four-test model and a new classification for the necropsy and SCT results, being positive only the samples with 100 or more *E. multilocularis*. In this case, the optimal cut-off point determined by the Bayesian mAb-ELISA ROC was still 0.16, conferring to the coproantigen test with 70.5% sensitivity, 80.0% specificity and an overall accuracy of 76.2% given by the AUC. Figure 7 shows the corresponding mAb-ELISA ROC curve.

## Discussion

The employment of latent class models to analyse the results of the diagnostic tests for *E. multilocularis* allowed the determination of the performance of the test in the study population and the estimation of the true parasite prevalence in the absence of a perfect gold standard test. Furthermore, it was also possible to adjust for potential conditional dependence between tests. Also, these models could evaluate the association between three covariates and parasite infection occurrence in the fox. Likewise, the application of latent class models permitted the building of ROC curves for the ELISAs following a Bayesian approach that enabled the empirical determination of the best cut-off point and the evaluation of the impact that the selection of the cut-off had in the estimation of the rest of the characteristics of the test.

In the present study, the latent class models including all potential covariances between sensitivities proved to be robust and their parameter estimates showed to be consistent with previous knowledge. The point estimates for the true *E. multilocularis* prevalence in foxes given by the three and four-test models (without covariates) were 58.4 and 59.5%, respectively. Similar high parasite prevalences have been previously reported in Swiss foxes [46–48]. In regard to the tests performances, the model estimates are also in line with prior information on diagnostics accuracy of these techniques. The best-fitted models (without covariates) gave high point estimates for the necropsy and SCT sensitivities, 91.9 and 88.5%. The SCT’s sensitivity has commonly been considered relatively high, 98–100% [38] since the immersion of the intestines in saline solution and the posterior scrapping of the intestinal wall ensures the release of most of the worms [36]. Hence, if a fox has intestinal worms this method should identify them reliably. However, an experimental study determined that intestinal samples should contain at least 10 tapeworms to achieve a 60% probability of obtaining positive detection [8]. Although experimental conditions differ from natural infection, this study highlights SCT’s sensitivity limitations related to worm burdens. In addition, the combination of worms’ degradation during post-mortem conditions plus the intestines deep-freezing stage involved in the SCT process could also affect the SCT’s sensitivity. Moreover, a recent latent class analysis of *E. multilocularis* diagnostic tests estimated the SCT-sensitivity to be between 76 and 88% [9]. Hence, SCT should not be regarded as a “gold standard” test [6]. The estimated specificities of the pAb-ELISA from the three and four-test models (without covariates) ranged between 54.0–73.2% and 55.8–75.6%, respectively. The estimated specificity of the mAb-ELISA was found amid 60.1–79.4%. Coproantigen specificities can be altered by the occurrence of cross-reactions with antigens from concomitant helminths infections [13] or even the persistence of *E. multilocularis* antigens in the faeces after the fox is no longer infected resulting in false positives results. The pAb-ELISA and the mAb-ELISA’s estimated sensitivities from the three and four-test models (without covariates) ranged between 47.5–63.7% and 48–63.9% for the pAb-ELISA and 55.3–70.8% for the mAb-ELISA. Coproantigen sensitivities strongly depend on the intensity of *E. multilocularis* infection [13, 17–19], so foxes with low worm burdens are more likely to result in false negatives. Knowing how highly aggregated distributed is *E. multilocularis* in the fox population, it is likely that some foxes harboring low worm burdens will be misclassified as negatives by this type of test. Overall, the best model showed that the mAb-ELISA performed slightly better than the pAb-ELISA. Our pAb-ELISA estimates are in line with a prior latent class study that included arecoline purgation and egg-PCR in their analysis (SEdog 55%, 95% CI: 40.8–68.9% and SPdog 70.6%, 95% CI: 65.3–76.7%) [35], but lower than the originally test characteristics reported (SEfox ~80%, SPfox 95–99%) [13]. Often the coproantigen test has been evaluated using the SCT as the gold standard test [13, 18] even though, as we have discussed previously, its sensitivity is not perfect. Taking this into account the coproantigen test’s actual sensitivity in the field can be realistically considered to be around 60% [6]. Furthermore, ELISA assays using polyclonal antibodies are prone to batch-to-batch variation and thus their performance reproducibility cannot be guaranteed. In this study however, sufficient quantities of polyclonal antibodies were produced in one batch to allow 400,000 tests, which could be the basis of minimizing this issue. In addition, the use of the polyclonal antibody test permitted the use of the three or four-test models and thus was important to help define the parameters of the other tests used, which do not suffer from this potential issue. Lastly, the estimates obtained from the three and four-test models for the egg-PCR specificities ranged between 85.0–98.2% and 87.3–99.1% and their sensitivities amid 47.4–61.0% and 48.5–61.0%. A field study in Kyrgyzstan also described the performance of this multiplex PCR as a highly specific but low sensitive test (SEdog 50%, 95% CI: 29–72% and SPdog 100%, 95% CI: 97–100%) [49]. High specificities are expected because the primers of this egg-PCR can identify and differentiate specifically the *Echinococcus* egg-DNA found in the faeces, even though there is always the possibility of false positive animals resulting from cross-contamination [50]. In general, the PCR’s sensitivity might be low under low worm burdens conditions or the presence of juvenile worms (characteristic during pre-patent infections) [25]. Furthermore, during the DNA isolation procedure PCR-inhibitory substances could be in the sample increasing the number of false negative results [25, 50].

In our models we considered conditional dependencies between sensitivities, but not specificities. The absence of evident covariances among specificities can at least partly be explained by the relatively high specificities and hence a low number of false positives resulting in a too small sample size to gain any information for these covariances.

In both analyses, including the data of three and four tests, two covariates were identified as significantly associated with *E. multilocularis* presence in the fox: age and concomitant infection with other cestodes. The incorporation of the effect of fox age and the co-infection with other cestodes improved the goodness-of-fit of the model to the data and did not alter the estimation of the accuracy of the diagnostic tests. Cestode species such as *E. multilocularis*, *Taenia* spp. or *Mesocestoides* spp. have been found in the intestine of the red fox in Switzerland [46, 51]. Furthermore, these tapeworms share with *E. multilocularis* the same species of rodents as intermediate hosts (i.e. *Microtus arvalis*, *Arvicola terrestris*) [46, 51]. Preying on rodents harbouring diverse species of cestodes results in co-infections in the definitive host. This supports the model finding of foxes with concomitant cestodes infection presenting double the odds of harbouring *E. multilocularis*. There are several studies relating foxes of a young age to *E. multilocularis* infection, although not always this difference has been found statistically significant [46, 48, 52]. Several hypotheses have been formulated to explain the frequent reporting of parasite infection and burdens in juvenile foxes. One of the most suggested reasons behind these age-differences is the potential existence of an acquired immunological response after repeated infection [46, 48, 53]. However, other plausible causes such as differences in their predatory or territorial behaviour might result in juvenile animals with higher exposure to *E. multilocularis* infection compared to adults [54, 55]. A recent study modelling *E. multilocularis* abundance in Zurich foxes suggests that variations in infection pressure among age groups might be behind the observed differences in parasite loads between juveniles and adults [56]. Nevertheless, in our study, the fox age was estimated on visual examination of teeth wear assessed by the researcher who was identifying the animals. Despite being a quick and easy method to distinguish between older and younger animals, it is also known to be less than 100% reliable as the teeth wear is subjected to individual characteristics such as the type of diet or the occurrence of missing teeth [39, 57]. There is less evidence that sustains the potential association between *E. multilocularis* infection and sex of the fox [58]. Although young male foxes are known to expand their territory during the mating season [59] and thus, might have a higher risk of infection if, during their roaming behaviour, they trespass clusters presenting an active parasite cycle with infected rodents. Nevertheless, the models did not find any significant differences in the odds of *E. multilocularis* infection between male and female foxes. This might be caused due to the small size of the study population or because of an unbalance of proportions in the data set, although the difference between numbers of collected males and females was not remarkable. Due to the small sample size, no internal validation was possible. Potentially, two sources of bias might have occurred. First, it could be that due to the sampling of the foxes during the hunting seasons a seasonal variation in cestode infection [56] might have introduced some sort of bias. Secondly, the PCR is designed to detect patent, but not pre-patent infections. With a life duration of 90 days and a third of this time being in a pre-patent state, the PCR results will never be unbiased in detecting all *E. multilocularis* infections [60].

For this analysis, uninformative as well as informative priors based on existing knowledge were used. By sensitivity analyses varying our prior information systematically, we found that our results are robust and are driven by the data and not by the prior information. Furthermore, the specificity of the necropsy and SCT was fixed at 100% [36]. Also, the assumption of a high specificity in the identification of parasites by necropsy and SCT is supported by the lack of a potential differential diagnosis as, to the authors’ knowledge, *E. granulosus* has not been yet found in foxes in Switzerland.

Here, we wanted to assess the difference in the determination of the cut-off by using two methods: the classical approach of considering the necropsy and SCT as a perfectly accurate test and the empirical method of deriving the ROC curve using the parameter estimations of the Bayesian latent class model. On this occasion, some differences were found, as the cut-off points obtained from the Bayesian methods were slightly higher than those obtained from the classical approach. To some extent, the use of the classic method of treating the necropsy and SCT results as true infection status to establish the coproantigen test accuracy could underestimate the specificity of the ELISA, in the case of having several necropsy and SCT false negatives. Also, the building of the Bayesian ROC curves proved that the variation in the selection of the cut-off point for the ELISA did not affect the estimations of the other tests when including just one ELISA in the analysis. When including the two ELISAs the selection of the mAb-ELISA cut-off point did have an impact only on the pAb-ELISA estimations as the model structure accounted for conditional dependency between both coproantigen tests.

Finally, we employed the Bayesian latent class models to evaluate the test accuracy of the monoclonal ELISA to identify foxes presenting high parasite burdens of 100 or more worms. The distribution of *E. multilocularis* in the fox population is highly aggregated with few animals making the largest contribution to the environmental contamination with parasitic eggs, and thus representing the majority of the zoonotic risk [21]. However, it is also possible that foxes with low worm burdens at the time of sampling could have had much higher burdens a short period before due to the dynamics of infection [60]. The highly infected foxes are believed to play a critical role in *E. multilocularis* transmission and ultimately human infection. Therefore, when monitoring this zoonotic parasite in the fox population, it is paramount that surveillance programs employ diagnostic tests that can identify foxes effectively harbouring high parasite loads. The monoclonal coproantigen test proved to be a good tool for this purpose, showing high sensitivity and specificity to identify animals with moderate-to-high parasite burdens (≥ 100 worms). Furthermore, its good test performance along with its economic implementation and the fact that it can be performed on the faecal field samples without the need to collect dead animals, make this diagnostic test suitable for population studies in endemic areas.

However, in low prevalence and free areas where both a high sensitivity and a very high specificity (close to 100%) are needed, a confirmatory test is required. Although the MC-PCR fulfils these requirements [30], it has to be ensured that sufficient material from the fox scat will be available for both tests to be performed on the collected faecal samples. Otherwise, the whole fox has to be collected, and the ELISA should be done on intestinal contents.

## Conclusions

Through the implementation of Bayesian latent class models, we could estimate the prevalence of infection and the specific performance of four diagnostic tests for *E. multilocularis* on the study population. As we have seen, there is a lack of a gold standard test for *E. multilocularis* diagnosis in the definitive host. Furthermore, we know that the performance of these diagnostic techniques varies depending on the population investigated. Thus, the particular test performance on the population investigated has to be accounted for to be able to correctly interpret the diagnosis results [61]. The adoption of a Bayesian latent class approach helps to overcome the absence of a perfectly accurate test and therefore gives a more reliable indication of the tests performance to ensure that meaningful conclusions can be drawn. Furthermore, the flexibility inherent in this type of models allows the incorporation of the potential dependence between diagnostic tests and permits the investigation of the association of potential risk factors with true disease status [35, 49]. Finally, in the case of using a diagnostic test that needs the establishment of a cut-off point for the interpretation of its results, the Bayesian modelling facilitates the selection of this threshold value more reliably and comprehensively than the classical method.

## Additional files

Additional file 1: Table S1. Original data file. (XLS 74 kb)

Additional file 2: Bayesian latent-class model code for three diagnostic tests. (DOC 33 kb)

Additional file 3: Bayesian latent-class model code for four diagnostic tests. (DOC 43 kb)

Additional file 4: Table S2. Description of the prior information used in the latent class models for three diagnostic tests. (DOC 35 kb)

Additional file 5: Table S3. Description of the prior information used in the latent class models for four diagnostic tests. (DOC 38 kb)

Additional file 6: The sensitivity analysis for the sensitivity of PCR. (PDF 320 kb)

Additional file 7: **Table S4**. Resulting covariances between sensitivities of the 3 and 4 test model. (DOCX 13 kb)

Additional file 8: Figure S1. Polyclonal ELISA ROC curves produced using the classical and the Bayesian approach (4 tests). (TIFF 501 kb)

Additional file 9: Figure S2. Monoclonal ELISA ROC curves produced using the classical and the Bayesian approach (4 tests). (TIFF 501 kb)

## Abbreviations

AE: Alveolar echinococcosis
DIC: Deviance information criterion
DNA: Deoxyribonucleic acid
ELISA: Enzyme-linked immunosorbent assay
mAb: Monoclonal antibodies
MCMC: Markov chain Monte Carlo
pAb: Polyclonal antibodies
ROC: Receiver operating characteristic
SCT: Sedimentation and counting technique
Se: Sensitivity
SLT: Sedimentation counting technique
Sp: Specificity
