# Supplementary material for: Latent class models for Echinococcus multilocularis diagnosis in foxes in Switzerland in the absence of a gold standard
Source: Parasit Vectors. 2017 Dec 19;10:612. doi: 10.1186/s13071-017-2562-1 (PMC5737983; doi:10.1186/s13071-017-2562-1)
Supplement: Supplementary file 2 — Bayesian latent-class model code for three diagnostic tests. (DOC 33 kb) [file 13071_2017_2562_MOESM2_ESM.doc]

**Additional file 2. Bayesian latent-class model code for three diagnostic tests**

#######################################################

##Definition of the variables in the model

#######################################################

var p[N], q[N,8], pr[N], L[N],checks[N,16];

#N <- observations (N=300 foxes)

# p <- individual samples

# q <- different combinations of test results

# pr <- prevalence

# s <- test sensitivities

# c <- test specificities

# cs <- conditional dependency between tests sensitivities

# cc <- conditional dependency between tests specificities

# m.short <- data set name

#######################################################

## Modelling the different probabilities of combinations of tests results

#######################################################

model {

for(i in 1:N){

q[i,1]<-pr[i]*(s1*s2*s3+cs12+cs13+cs23)+(1-pr[i])*((1-c1)*(1-c2)*(1-c3)+cc12+cc13+cc23);

q[i,2]<-pr[i]*(s1*s2*(1-s3)+cs12-cs13-cs23)+(1-pr[i])*((1-c1)*(1-c2)*c3+cc12-cc13-cc23);

q[i,3]<-pr[i]*(s1*(1-s2)*s3-cs12+cs13-cs23)+(1-pr[i])*((1-c1)*c2*(1-c3)-cc12+cc13-cc23);

q[i,4]<-pr[i]*(s1*(1-s2)*(1-s3)-cs12-cs13+cs23)+(1-pr[i])*((1-c1)*c2*c3-cc12-cc13+cc23);

q[i,5]<-pr[i]*((1-s1)*s2*s3-cs12-cs13+cs23)+(1-pr[i])*(c1*(1-c2)*(1-c3)-cc12-cc13+cc23);

q[i,6]<-pr[i]*((1-s1)*s2*(1-s3)-cs12+cs13-cs23)+(1-pr[i])*(c1*(1-c2)*c3-cc12+cc13-cc23);

q[i,7]<-pr[i]*((1-s1)*(1-s2)*s3+cs12-cs13-cs23)+(1-pr[i])*(c1*c2*(1-c3)+cc12-cc13-cc23);

q[i,8]<-pr[i]*((1-s1)*(1-s2)*(1-s3)+cs12+cs13+cs23)+(1-pr[i])*(c1*c2*c3+cc12+cc13+cc23);

#######################################################

## Check and correct potential errors of probabilities exceeding (0,1) bounds

#######################################################

checks[i,1]<- s1*s2*s3+cs12+cs13+cs23;

checks[i,2]<- (1-c1)*(1-c2)*(1-c3)+cc12+cc13+cc23;

checks[i,3]<- s1*s2*(1-s3)+cs12-cs13-cs23;

checks[i,4]<- (1-c1)*(1-c2)*c3+cc12-cc13-cc23;

checks[i,5]<- s1*(1-s2)*s3-cs12+cs13-cs23;

checks[i,6]<- (1-c1)*c2*(1-c3)-cc12+cc13-cc23;

checks[i,7]<- s1*(1-s2)*(1-s3)-cs12-cs13+cs23;

checks[i,8]<- (1-c1)*c2*c3-cc12-cc13+cc23;

checks[i,9]<- (1-s1)*s2*s3-cs12-cs13+cs23;

checks[i,10]<- c1*(1-c2)*(1-c3)-cc12-cc13+cc23;

checks[i,11]<- (1-s1)*s2*(1-s3)-cs12+cs13-cs23;

checks[i,12]<- c1*(1-c2)*c3-cc12+cc13-cc23;

checks[i,13]<- (1-s1)*(1-s2)*s3+cs12-cs13-cs23;

checks[i,14]<- c1*c2*(1-c3)+cc12-cc13-cc23;

checks[i,15]<- (1-s1)*(1-s2)*(1-s3)+cs12+cs13+cs23;

checks[i,16]<- c1*c2*c3+cc12+cc13+cc23;

valid[i]<- step(1-q[i,1])*step(q[i,1])*

step(1-q[i,2])*step(q[i,2])*

step(1-q[i,3])*step(q[i,3])*

step(1-q[i,4])*step(q[i,4])*

step(1-q[i,5])*step(q[i,5])*

step(1-q[i,6])*step(q[i,6])*

step(1-q[i,7])*step(q[i,7])*

step(1-q[i,8])*step(q[i,8])*

step(1-checks[i,1])*step(checks[i,1])*

step(1-checks[i,2])*step(checks[i,2])*

step(1-checks[i,3])*step(checks[i,3])*

step(1-checks[i,4])*step(checks[i,4])*

step(1-checks[i,5])*step(checks[i,5])*

step(1-checks[i,6])*step(checks[i,6])*

step(1-checks[i,7])*step(checks[i,7])*

step(1-checks[i,8])*step(checks[i,8])*

step(1-checks[i,9])*step(checks[i,9])*

step(1-checks[i,10])*step(checks[i,10])*

step(1-checks[i,11])*step(checks[i,11])*

step(1-checks[i,12])*step(checks[i,12])*

step(1-checks[i,13])*step(checks[i,13])*

step(1-checks[i,14])*step(checks[i,14])*

step(1-checks[i,15])*step(checks[i,15])*

step(1-checks[i,16])*step(checks[i,16]);

#######################################################

## Contribution to the likelihood for each observation

#######################################################

L[i]<- equals(valid[i],1)*(

equals(m.short [i,1],1)*equals(m.short[i,2],1)*equals(m.short [i,3],1)*q[i,1]

+ equals(m.short [i,1],1)*equals(m.short[i,2],1)*equals(m.short [i,3],0)*q[i,2]

+ equals(m.short [i,1],1)*equals(m.short[i,2],0)*equals(m.short [i,3],1)*q[i,3]

+ equals(m.short [i,1],1)*equals(m.short[i,2],0)*equals(m.short [i,3],0)*q[i,4]

+ equals(m.short [i,1],0)*equals(m.short[i,2],1)*equals(m.short [i,3],1)*q[i,5]

+ equals(m.short [i,1],0)*equals(m.short[i,2],1)*equals(m.short [i,3],0)*q[i,6]

+ equals(m.short [i,1],0)*equals(m.short[i,2],0)*equals(m.short [i,3],1)*q[i,7]

+ equals(m.short [i,1],0)*equals(m.short[i,2],0)*equals(m.short [i,3],0)*q[i,8]

) +(1-equals(valid[i],1)) *(1e-14);

## When adding covariates to the model

logit(pr[i])<-intercept+slope*m.short[i,6];

##Without covariates:

pr[i]<-prc

#######################################################

## Trick to ensure the probabilities are always less than 1

#######################################################

p[i] <- L[i] / 1;## divided by a constant just to ensure all p's <1

ones[i] ~ dbern(p[i]);

}

#######################################################

## Definition of model priors

#######################################################

## Covariance terms

covs12~dunif(-1,1);

covs13~dunif(-1,1);

covs23~dunif(-1,1);

covc12<-0;

covc13<-0;

covc23<-0;

prc~dbeta(37.9836,31.2593); # Prevalence

c1<-1; # SP necropsy

c2~dbeta(1,1); # SP PCR

c3~dbeta(1,1); # SP ELISA pab

s1~dbeta(99.6983,6.1946); # SE necropsy

s2~dbeta(37.9836,31.2593); # SE PCR

s3~dbeta(1,1); # SE ELISA pab

logL<-sum(log(p[1:N]));

## When adding covariates to the model

#intercept~dnorm(0,0.001);

#slope~dnorm(0,0.001);

logL<-sum(log(p[1:N]));

}
