# Supplementary material for: Latent class models for Echinococcus multilocularis diagnosis in foxes in Switzerland in the absence of a gold standard
Source: Parasit Vectors. 2017 Dec 19;10:612. doi: 10.1186/s13071-017-2562-1 (PMC5737983; doi:10.1186/s13071-017-2562-1)
Supplement: Supplementary file 5 — Description of the prior information used in the latent class models for four diagnostic tests. (DOC 38 kb) [file 13071_2017_2562_MOESM5_ESM.doc]

**Additional file 5. Table S3. Description of the prior information used in the latent class models for four diagnostic tests**

| **Parameters** |  | **Distribution** |  |
| --- | --- | --- | --- |
|  |  | **dunif()** |  |
| Covariances | Sensitivities | (-1,1) |  |
| Covariances | Specificity | - | Fixed to 0 |
|  | | | |
|  | | **dbeta(a,b)** |  |
| Necropsy | Sensitivity | (99.6983,6.1946)^a^ |  |
|  | Specificity | - | Fixed to 1 |
| Egg-PCR | Sensitivity | (37.9836,31.2593)^b^ |  |
|  | Specificity | (36.7028,2.8791)^c^ |  |
| pAb-ELISA | Sensitivity | (1,1) |  |
|  | Specificity | (1,1) |  |
| mAb-ELISA | Sensitivity | (1,1) |  |
|  | Specificity | (1,1) |  |
| Prevalence | | (37.9836,31.2593)^d^ |  |

All informative priors were obtained by using betabuster (<http://cadms.ucdavis.edu/diagnostictests/betabuster.html>), entering the following

^a^ Being 95% sure, that the sensitivity of necropsy is larger than 0.9 with a mode at 0.95

^b^ Being 95% sure, that the sensitivity of egg-PCR is larger than 0.45 with a mode at 0.55

^c^ Being 95% sure, that the specificity of egg-PCR is larger than 0.85 with a mode at 0.95

^d^ Being 95% sure, that the prevalence is larger than 0.45 with a mode at 0.55
