# Supplementary material for: Latent class models for Echinococcus multilocularis diagnosis in foxes in Switzerland in the absence of a gold standard
Source: Parasit Vectors. 2017 Dec 19;10:612. doi: 10.1186/s13071-017-2562-1 (PMC5737983; doi:10.1186/s13071-017-2562-1)
Supplement: Supplementary file 6 — The sensitivity analysis for the sensitivity of PCR. (PDF 320 kb) [file 13071_2017_2562_MOESM6_ESM.pdf]

Supplementary sensitivity analysis (PCR)

July 2017

Sensitivity analysis to assess the robustness of the Bayesian latent class analysis:

We used informative priors, based on literature, for prevalence, sensitivity of necropsy, sensitivity and specificity of PCR. Values for the specific beta distributions were obtained with the program betabuster http://cadms.ucdavis.edu/diagnostictests/betabuster.html.

- 1) Constant informative prior on prevalence: dbeta(37.9836, 31.2593) obtained by betabuster "being 95% sure, that the prevalence is larger than 0.45 with a mode at 0.55".
- 2) Constant informative prior on sensitivity of necropsy and subsequent SCT: dbeta(99.6983,6.1946) obtained by betabuster "being 95% sure, that the sensitivity of necropsy/SCT is larger than 0.9 with a mode at 0.95".

We varied the informative prior for the sensitivity of the PCR systematically from assuming that the sensitivity is larger than 0.9, 0.8 and so on until 0.1, with a respective mode of 0.95, 0.85 and so on until 0.25. With this approach we obtained a number of informative priors, ranging from strong priors with a small variance (steep curve) or high precision, e.g. "greater than 60 % with a mode at 65%" to rather uninformative priors e.g. "greater than 10% and a mode at 95%" (flat curve). The latter one is close to the independence model with priors dbeta(1,1). Furthermore with this approach we also obtained a number of priors which are -potentially- in conflict with our data, e.g. we assume that the sensitivity is not close to 95% or 25%.

The idea was (in line with Jim Albert in "Bayesian computation with R", 2nd ed., 2009 Springer on page 45 "[. . .] where different priors are possible, it is desirable that inferences from the posterior not to be dependent from the exact functional form of the prior. A Bayesian analysis is said to be robust to the choice of prior if the inference is insensitive to different priors that match the user's belief."

We deliberately chose a number of informative priors which match our prior beliefs by successively allowing a wider range of the prior to assess the prior's influence on the posterior density distribution. Although Bayesian analysis allows for incorporating "subjective" prior information (which might be useful in some cases) in our analysis we wanted the data (or the likelihood thereof) to be the main drivers for the posteriors. Thus we expected to see - for priors in disagreement with the data - a different posterior distribution compared to the posteriors of our model in the main paper and this difference being more pronounced with stronger priors.

When looking at the 44 models run with different informative priors consecutively, a clear pattern for the sensitivity of PCR - which we varied systematically - becomes clear. A similar pattern although considerably less pronounced is also present in the five other parameters of interest. For the posterior sensitivities of PCR, the models 1,10,18,25 are clearly above and the models 36, 40 and 45 are clearly below the estimates when informative priors in agreement with existing knowledge. All these models have in common that relatively strong priors (steep curve) due to assuming that "we are 95% sure that the sensitivity of PCR is larger than 0.9 with a mode of 0.95", ". . . larger than 0.8 with a mode of 0.85" and so on in steps of 0.1 until ". . . larger than 0.2 with a mode of 0.25". The sum of a and b for this model is also approx. 50% of the sample size, indicating also the high, presumably too high prior strength for this data set.

In the following first, the means of the posteriors for the prevalence and each of the test accuracies estimated in the three-test model in the 44 models are shown. Then, for purpose of illustration, the densities for the first model (out of 44), including the prior as well as the posterior densities with informative priors and covariances as well as under independence assumption.

In a similar way - we also run sensitivity analyses for varying the sensitivity of necropsy/SCT and prevalence (not shown) - we believe that our results are robust, i.e. not influenced by the informative priors.

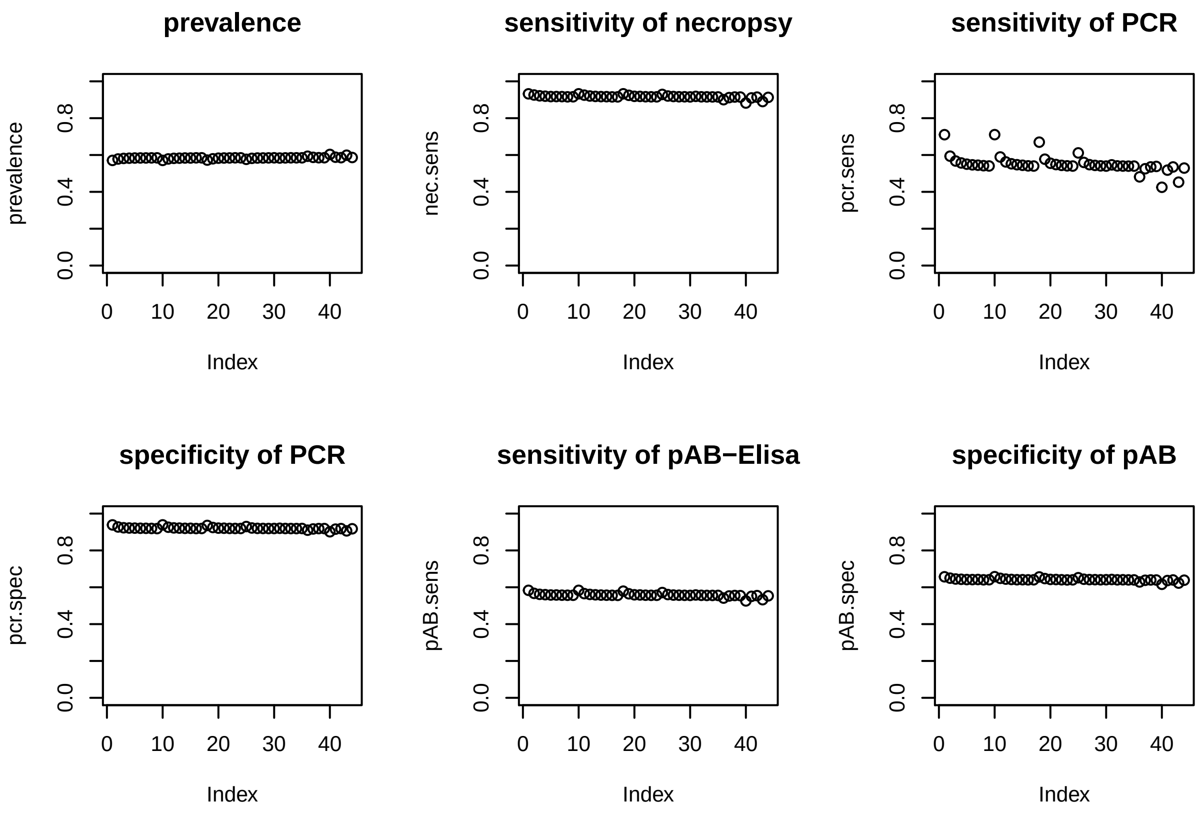
prevalence
prevalence
Index
sensitivity of necropsy
nec.sens
Index
sensitivity of PCR
pcr.sens
Index
specificity of PCR
pcr.spec
Index
sensitivity of pAB−Elisa
pAB.sens
Index
specificity of pAB
pAB.spec
Index
0.0
0.4
0.8
0
10
20
30
40

1) density plots for prior for sensitivity of PCR beta(99.6983, 6.1946)

meaning: 95 % sure, that se of PCR is greater than 0.9 with a mode at 0.95.

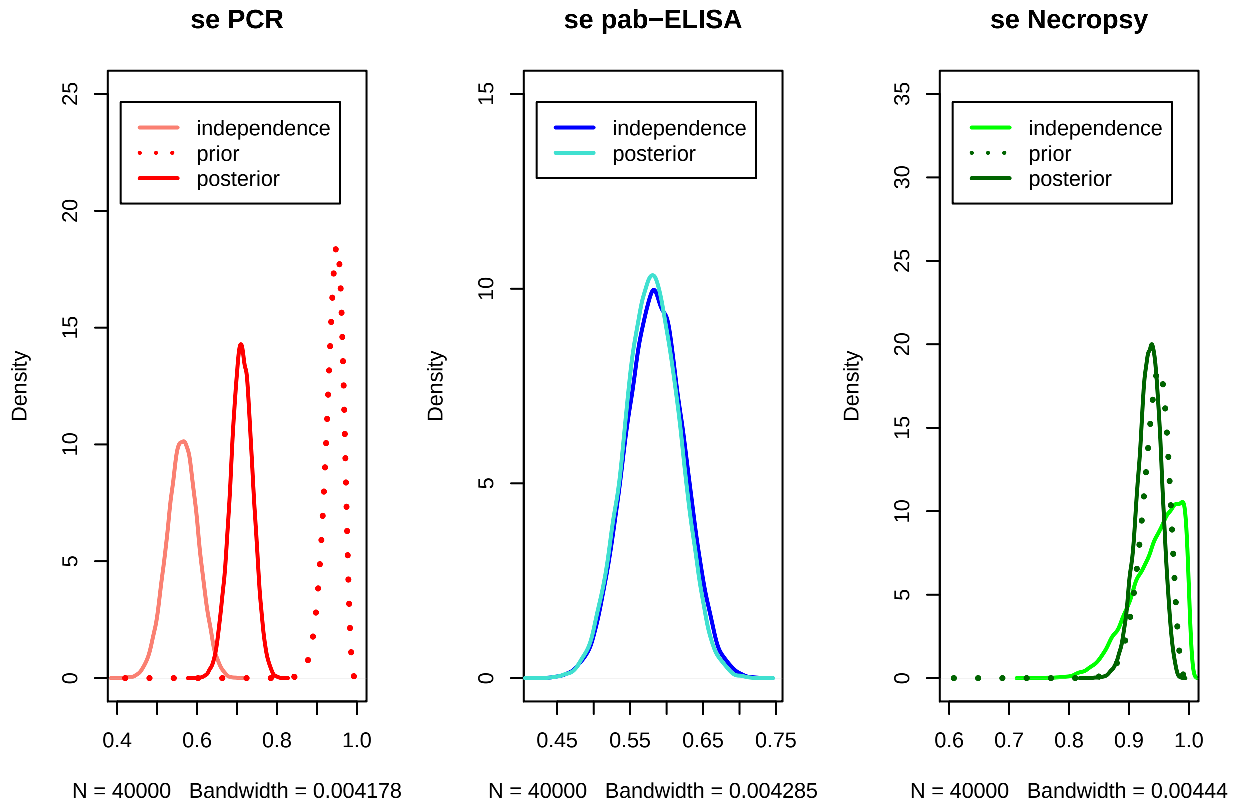

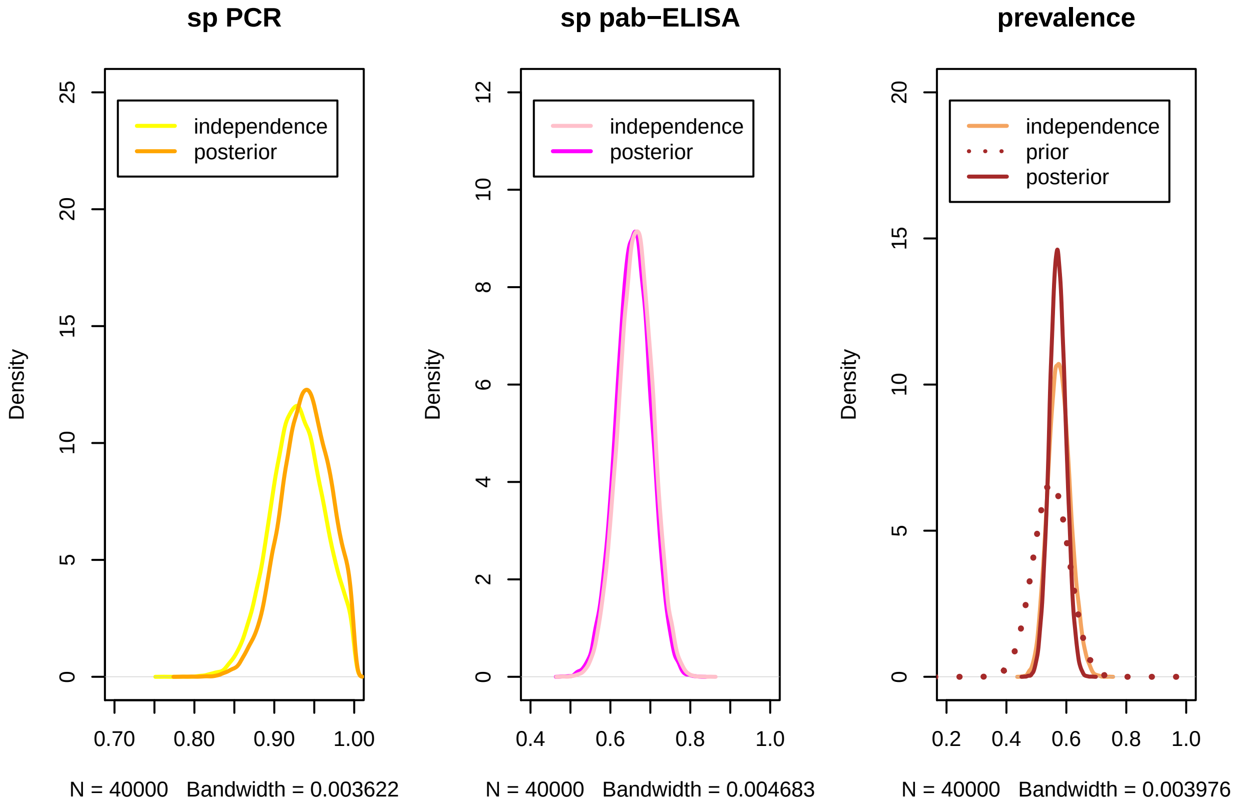

sp PCR
independence
posterior
Density
0
5
10
15
20
25
0.70
0.80
0.90
1.00
N = 40000 Bandwidth = 0.003622
sp pab−ELISA
independence
posterior
Density
0
2
4
6
8
10
12
0.4
0.6
0.8
1.0
N = 40000 Bandwidth = 0.004683
prevalence
independence
prior
posterior
Density
0
5
10
15
20
0.2
0.4
0.6
0.8
1.0
N = 40000 Bandwidth = 0.003976

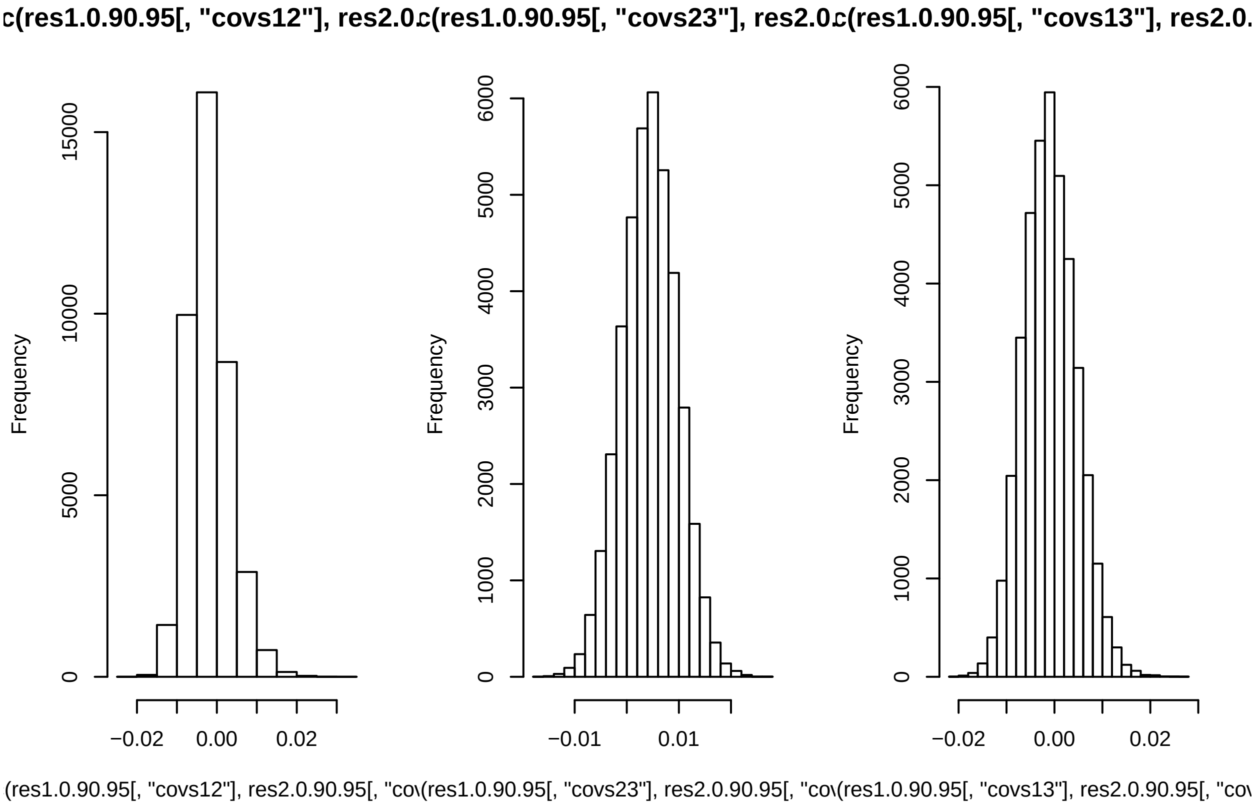
c(res1.0.90.95[, "covs12"], res2.0.
c(res1.0.90.95[, "covs23"], res2.0.
c(res1.0.90.95[, "covs13"], res2.0.
Frequency
0
5000
10000
15000
−0.02
0.00
0.02
1000
2000
3000
4000
5000
6000
−0.01
0.01
(res1.0.90.95[, "covs12"], res2.0.90.95[, "cov
(res1.0.90.95[, "covs23"], res2.0.90.95[, "cov
(res1.0.90.95[, "covs13"], res2.0.90.95[, "cov
