# Supplementary material for: Latent class models for Echinococcus multilocularis diagnosis in foxes in Switzerland in the absence of a gold standard
Source: Parasit Vectors. 2017 Dec 19;10:612. doi: 10.1186/s13071-017-2562-1 (PMC5737983; doi:10.1186/s13071-017-2562-1)
Supplement: Supplementary file 7 — Table S4. Resulting covariances between sensitivities of the 3 and 4 test model. (DOCX 13 kb) [file 13071_2017_2562_MOESM7_ESM.docx]

**Additional file 7. Table S4. Resulting covariances between sensitivities of the 3 and 4 test model**

| **Covariance between sensitivities of** | **3 test model** | **4 test model** |
| --- | --- | --- |
| Necropsy SCT and PCR | 0.0086 | 0.0021 |
| Necropsy SCT and pAb-Elisa | 0.0100 | 0.0019 |
| PCR and pAb-Elisa | 0.0094 | 0.0042 |
| Necropsy SCT and mAb-Elisa | NA^c^ | 0.0033 |
| PCR and mAb-Elisa | NA^c^ | 0.0043 |
| pAb-Elisa and mAb-Elisa | NA^c^ | 0.0077 |

NA^c^: not applicable
