# Supplementary figures and images for: Latent class models for Echinococcus multilocularis diagnosis in foxes in Switzerland in the absence of a gold standard
Source: Parasit Vectors. 2017 Dec 19;10:612. doi: 10.1186/s13071-017-2562-1 (PMC5737983; doi:10.1186/s13071-017-2562-1)

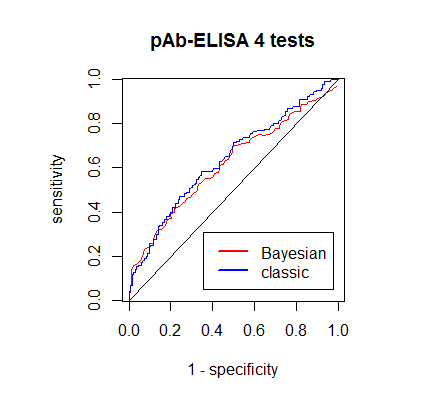

Supplement: Supplementary file 8 — Polyclonal ELISA ROC curves produced using the classical and the Bayesian approach (4 tests). (TIFF 501 kb) [file 13071_2017_2562_MOESM8_ESM.tiff]

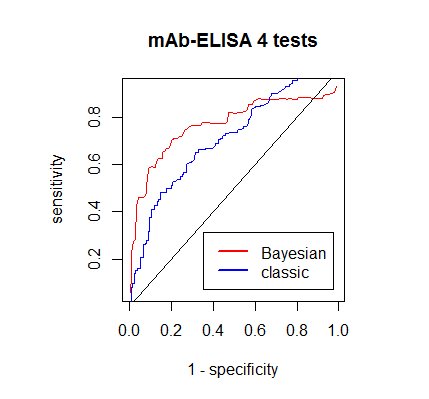

Supplement: Supplementary file 9 — Monoclonal ELISA ROC curves produced using the classical and the Bayesian approach (4 tests). (TIFF 501 kb) [file 13071_2017_2562_MOESM9_ESM.tiff]
